# Implementation of "social and communicative competencies" in medical education. The importance of curriculum, organisational and human resource development

**DOI:** 10.3205/zma000992

**Published:** 2015-11-16

**Authors:** Susanne Pruskil, Nicole Deis, Susanne Druener, Claudia Kiessling, Swetlana Philipp, Katrin Rockenbauch

**Affiliations:** 1 Freie und Hansestadt Hamburg, Gesundheitsamt Altona, Deutschland; 2 Medizinische Fakultät Mannheim der Universität Heidelberg, Geschäftsbereich Studium und Lehrentwicklung, Mannheim, Deutschland; 3 RWTH Aachen, Universitätsklinikum, Medizinische Fakultät, Skillslab, Aachener Interdisziplinäres Trainingszentrum für medizinische Ausbildung, Aachen, Deutschland; 4 Medizinische Hochschule Brandenburg Theodor Fontane, Bereich Assessment und Prüfungsorganisation, Neuruppin, Deutschland; 5 Uniklinikum Friedrich-Schiller-Universität Jena, Institut für Psychosoziale Medizin und Psychotherapie, Jena, Deutschland; 6 Universitätsklinikum Leipzig AöR, Department für Psychische Gesundheit, Abteilung für Medizinische Psychologie und Medizinische Soziologie, Leipzig, Deutschland

**Keywords:** Communicative and social competencies, medical education, curriculum development, organisational development, human resource development

## Abstract

**Objective:** With this article we want to support teachers and curriculum planners to be aware of and apply knowledge and recommendations of organisational (OD), curriculums (CD) and human resource development (HRD) ideas already in the planning phase of a project. Taking these into account can influence the process of change successfully and controlled during the introduction and establishment of curricula in the field of communication and social skills in medical education.

**Approach and Results:** In the context of a multi-stage developmental process, a recommendation on CD for "Communicative and social competencies" was developed. The basis for it was made during two workshops of the GMA-committee "Communicative and social competencies" and supplemented by the available literature and the experience of communication experts. The "Undeloher Recommendation" (see attachment ) includes a compilation of recommendations and guiding questions, which is geared to the various phases of CD. Additionally, general approaches and recommendations of organisational and human resource development were integrated, which turned out to be particularly relevant in the process of CD. Thus, the "Undeloher recommendation" includes an orientation for each phase of the curriculum development process, the organisation and the staff in order to successfully implement a longitudinal curriculum. In addition to theoretical models the long-term discussion process and the personal experiences of a variety of curriculum planners and teachers have been integrated.

**Conclusion: **The "Undeloher recommendation" can support the implementation processes of curricula in communication and social skills during development and realisation. Its application was reviewed in the context of workshops based on concrete examples. The participating teachers and curriculum planners assessed it to be very helpful. The recommendation goes beyond of what has been described in terms of content models in the CD so fare. In particular, the organisational and human resource development related aspects such as the formation of a steering committee and recommendations for the phase of sustainability.

## Zusammenfassung

**Zielsetzung:** Ziel dieser Arbeit ist es, Lehrenden und Curriculumsplanern die Bedeutung der Curriculums- (CE), Personal- (PE) und Organisationsentwicklung (OE), bereits in der Planungsphase eines Projekts aufzuzeigen. Diese können dann gezielt genutzt werden um Veränderungsprozesse bei der Einführung und Etablierung von Curricula im Bereich kommunikativer und sozialer Kompetenzen im Medizinstudium erfolgreich zu beeinflussen und zu steuern.

**Vorgehen und Ergebnisse:** Im Rahmen eines mehrstufigen Entwicklungsprozesses, in dem insbesondere zwei interfakultäre Workshops des GMA-Ausschusses „Kommunikative und soziale Kompetenzen“ im Zentrum standen, wurde aufbauend auf die verfügbare Literatur und den Erfahrungen von Kommunikationsexperten eine Empfehlung zur CE im Bereich „Kommunikativer und sozialer Kompetenzen“ erarbeitet. Die „Undeloher-Empfehlung“ (siehe Anhang ) beinhaltet eine Zusammenstellung von Empfehlungen und Leitfragen, die sich an den verschiedenen Phasen der CE orientiert. Zusätzlich wurden allgemeine Herangehensweisen und Empfehlungen der Organisations- und Personalorganisation integriert, die sich im Prozess der CE als besonders relevant herausstellten. Damit beinhaltet die „Undeloher-Empfehlung“ für jede einzelne Phase der Curriculumsentwicklung eine Orientierung, was in bezug auf das Curriculum, die Organisation und das Personal für eine erfolgreiche Implementierung von longitudinalen Curricula zu beachten ist. Neben theoretischen Modellen fließen insbesondere der langjährige Diskussionsprozess und die persönlichen Erfahrungen einer Vielzahl von Curriculumsplanern und Lehrenden ein.

**Fazit: **Die „Undeloher-Empfehlung“ kann die Vorbereitung und Begleitung von Implementierungsprozessen von Curricula im Bereich kommunikative und soziale Kompetenzen unterstützen. Ihre Anwendung wurde im Rahmen der Workshops auf konkrete Beispiele überprüft und wurde von den beteiligten Lehrenden und Curriculumsplanern als sehr hilfreich eingeschätzt. Sie geht insbesondere mit organisations- und personalbezogenen Aspekten wie der Bildung einer Steuerungsgruppe und Empfehlungen für die Phase der Verstetigung über das hinaus, was bisher inhaltlich an Modellen in der CE beschrieben wurde.

## Background

The importance of social and communicative competencies for the medical profession has been increasingly recognised in recent years. This identified the need to teach social and communicative competencies already during undergraduate medical school. Many international catalogues of learning objectives have been compiled including those requirements and defining the communication skills as a core competence, medical students must acquire during their studies [1], [2], [3]. In German-speaking countries a number of projects for the implementation of study modules in the field of communication and social skills has been developed in the last 15 years. A 2010 cross-sectional survey revealed that at 31 of the 32 participating medical schools, communication skills training was part of the curriculum in the German-speaking countries [4]. In 2011 and 2012 at the Annual Meetings of the Society for Medical Education (GMA) around 40 research and teaching projects on the subject "Communicative and social skills" were presented respectively. The exchange of teaching experiences emphasised great interest in the subject and successful implementation of educational projects. At the same time it revealed that projects were discontinued after a few years, despite good concepts and committed teachers. In retrospect, it is often less a matter of content or didactics, rather strategic aspects that determine the success or failure of a project, such as early involvement of decision-makers or the financing of the project.

Despite the extensive literature on how change processes can be successfully designed [5], [6], [7], concrete application of these recommendations is missing. It is a challenge for teachers who want to implement innovative teaching projects to act strategic and appropriate within the university's policy.

## Objectives

With this article we want to support teachers and curriculum planners to be aware of and apply knowledge and recommendations of organisational (OD), curriculums- (CD) and human resource development (HRD) early in the planning phase of a project. This gives a strategic advantage to successfully influence the change processes for the implementation of a curriculum in communication and social competencies in medical education.

The recommendations address all teachers who are involved in curriculum development in medical education regardless whether they aim for a longitudinal curriculum or to strengthen the teaching of communication and social skills in the form of a longitudinal section of the curriculum.

The first part of the paper provides an introduction to the topic of curriculum development (CD), which leads to a concrete guideline for use in practice - called "Undeloher Recommendation". This is followed by a deepening debate of organisational-(OD) and human resource development (HRD) issues which turned out to be particularly relevant in the process.

## Development Process

The "Undeloher Recommendation" was developed by members of the GMA committee "communicative and social competences" during a two-day workshop in Undeloh in 2008. The aim of the workshop was the development of “recommendations for a longitudinal curriculum for communicative and social competencies in undergraduate medical education.” 30 people from 13 medical schools in the German-speaking countries attended the workshop. All participants had experiences in the planning and implementation of teaching and/or testing communicative and/or social competencies. Three parallel groups worked on different issues some of which have already been published [8]. One of the groups dealt with conditions for CD and Change Management. It turned out that all participants had experienced difficulties and setbacks in the CD. Those experiences gave the impetus to develop recommendations for an effective implementation in the sense of a successful change management. According to the recommendations of the Association of the Scientific Medical Societies in Germany (AWMF) the development process of the guideline was moderated based on the nominal group technique [9], [10].

In this gradual process, all relevant areas identified by the group members were collected, divided into clusters and then weighted. A set of recommendations and key questions based on the different phases of the CE were generated. In a second step this compilation was revised on the basis of the existing literature [5], [6], [7], [11], [12], [13], [14], [15] followed by a discussion with the workshop participants. To test its applicability the revised version was applied during a workshop at the annual meeting of the GMA in Bochum 2010 [16] followed by another workshop during the GMA Conference held in Aachen in 2012. The aim of this workshop was to introduce instruments and theories for implementation of longitudinal curricula in the field of communicative and social competencies and to discuss and re-assess the challenges of the implementation process [17].

## Curriculum Development

The structuring of curricula today is less input or process-oriented, and more competence-based, e.g. outcome-oriented [18], [19]. The benefits of competence-oriented curricula are that on the one hand they represent a clear control instrument that sets the benchmark of achievement and helps avoid the fragmentation of teaching. Secondly, they facilitate the establishment of individual learning goals and give both students and teachers an outline of the desired result. Two models seem helpful to describe the individual steps in CD: Kern et al. (see Table 1 (Tab. 1)) describes in six steps a method or approach for effective and long-term establishment of a structured educational experience ("planned educational experience" Kern page 1) [13]. This applies both to individual seminars on a particular subject as well as multi-year courses or complete training programs. The individual steps are to run through again and relate to each other.

Thumser et al. lay a slightly different focus [19]. Their seven-steps model for CD describes among other things concrete methods that can be used in CE (see Table 2 (Tab. 2)).

## Results: the "Undeloher recommendations" for Curriculum Development

The following recommendations were developed by a multidisciplinary group of experts from different universities. The recommendations describe the different phases of the implementation process. Each phase requires different strategies. For each phase recommendations were collected and concretised with one or more key questions. The aim of the key questions is to assist the reader with finding specific and individual responses. The recommendation can be used as a checklist and can be adapted to the particular circumstances. Though specific individual phases or single key questions can be selected. It also includes references to the previously described theories on CD. The scope of OD and HRD will be described and discussed in a subsequent paragraph.

The "Undeloher recommendation" (see attachment ) builds on the ideas and concepts of OD and HRD. Some aspects we delve important will be described in more detail in the following section. 

## Organisational Development

Although it has long been a controversial issue to regard universities as organisations, this perspective seems to enforce [20]. According to the definition by Kieser and Kubicek organisations are social entities, which pursue certain goals and thereby have formal arrangements. The activities of the members should be aligned with the goals [21]. OD as a specific kind of change management focuses on interventions within organisations. However, change is not the only goal, but to strengthen the learning ability of the organisation [22]. OD refers to four areas:

1. quality and quantity of the organisation´s achievements and products,
2. staff who are competent and willing to learn,
3. adapted structures and processes
4. consolidated cooperation relationships [23]

Below we introduce two selected concepts of OD, which have proven valuable in the development and planning of curricula and have been referenced in the "Undeloher recommendation".

### 1. SWOT analysis (Strengths, Weaknesses, Opportunities, Threats)

In the 1960s the SWOT analysis was developed in the United States [24], [25]. It is now a tool widely used in project planning and project management. Considering the strength-weakness-dimension (Strengths - Weaknesses), the focus lies on factors determined by the organisation that is the faculty or department, which can be directly influenced. In the case of a longitudinal communication curriculum for instance the skills and interests of the teachers for the subject could be classified on this dimension. In contrast the opportunity-risk dimension (Opportunities - Threats) focuses on external factors beyond the control of the faculty. For example, to change the medical licensing regulations: to anchor communication skills as a teaching and assessment matter would provide a great opportunity for all curriculum developers in this area.

#### 2. Stakeholder Analysis

Stakeholder analysis is a method of risk management. Stakeholders of a project are all those who have a legitimate interest in the course or result of a process or project [22]. The following individuals and groups could assist the developing process of a longitudinal communication skills curriculum as stakeholder: the dean, the dean of student affairs, teachers, clinic directors, communication skills experts, possibly an existing simulated patient program, the student council or in general all students, and if necessary, the human resource department at the university hospital. Who to consider in practice, depends on the particular situation of the respective faculty. To identify stakeholders it has been found helpful to ask e.g.:

- who promotes the project?
- Who has got additional knowledge that could be useful for the project?
- Who could hinder or slow down the project?
- Who is required to collaborate? [26]

Once the stakeholders are found, it is important to identify their goals and expectations, such as: fear of additional workload, expectations of appropriate compensation for additional expenses, etc.. The next step will assess the stakeholders: what is their attitude towards the project – will the respective group of stakeholders promote or hinder of the project? What is their influence - low, medium or high? From there strategies for dealing with those involved can be derived. A person with high commitment and strong influence on others can be very useful for the project hence the person or group should be highly involved. For stakeholders with medium to high impact but little commitment to the project, it is important to find a motive customised approach.

As said in the "Undeloher recommendation", two support measures have proven particularly useful in the context of OD in order to ensure the success of projects: the establishment of a coordination group and, if it seems helpful, the use of external advisers (such as expert advice, facilitation, public relations).

The coordination group arranges the exchange between the formal hierarchical levels and the development process. Applied to the situation at medical schools the possibility of setting up a working group should include participants from different areas. The members of the working group should at best be cross-linked, with the clinical subjects and the various stakeholders (e.g. faculty). This guarantees a faculty-wide exchange, which creates awareness of the issues of the working group.

Depending on the results of the stakeholder analysis and the position and reputation of the coordination group within the curriculum development, it may be useful to call in an external adviser. This could be for example a person with expert status in the field of teaching communicative skills. He/she can contribute personal experiences during the curriculum development process.

## Human resource development

While developing a communication curriculum or individual courses sooner or later the question arises as to the appropriate qualification of teachers. For those who participate in the teaching and training process of the communication curriculum HRD methods may be required in order to convey the teaching skills that are necessary for teaching the specific content. For example, the skills necessary to facilitate teaching units with standardised patients, so that students benefit from teaching session. Furthermore, communication skills should not only be taught, but also assessed. Examiners must be able to evaluate in addition to the technical content the communication behaviour of the students. For this, the examiners must be familiar with the teaching concepts in order to assess the extent the students used purposefully the learned techniques in an examination. In turn train the trainer staff is necessary to put teachers in a position to teach and assess communication skills. This too must be planned at an early stage of a successful HRD.

The concepts and techniques applied in the communication curriculum should ideally be familiar to all teachers of the faculty and all practising physicians of the connected hospitals. Teaching of medical interviewing is not a mere teaching content. It is applied in primary and secondary care everyday. Physicians function as role models e.g. during bedside teaching and clinical placements. Whenever the communication strategies of clinicians do not match with the teaching students will get into a cognitive conflict. If in doubt, they will decide on the sort of communication strategies they are experiencing in every day clinical practise, no matter how well the teaching lessons are [12].

A comprehensive communication training covering the content of the communication curriculum is therefore a key factor for success. Otherwise students respond to the discrepancy between reality and the communication training and assign the learned content to the world of theory. To achieve sustainability, a combination of training and coaching methods in close cooperation between the curriculum supervisor and the local HRD department should be offered.

## Discussion

A successful CD, not only in the field of communication and social skills is complex and is always related to the organisation and its staff.

The “Undeloher recommendation” goes in particular with point 1 "generating a steering committee" and section 7 "sustainability" beyond what has been described in terms of content models in the CD and could therefore, be particularly useful for future projects.

The workshop on "Longitudinal Communication Curriculum" in 2012 in Aachen [27] focused on a theoretical deepening regarding tools and theories in the field of CD. And again the results showed that a common mistake in CD seems to be the disregard of the areas of OD and HRD. The conclusion of the workshop was that a reform or complete development of a new curriculum not only changed the curriculum, but also implies and demands changes in the organisation and its staff. That is, to implement and sustainably anchor a curriculum, it also needs appropriate personnel, to sustain the organisation and the conduction of the training. Therefore, dedicated staff must be equipped with expertise and adequate time resources and be motivated to carry out the tasks attributed in high quality. Furthermore, the organisational structures must allow members of staff to act reasonable. To know and understand the structures of the organisation is therefore of utmost importance in order to make good teaching and learning possible. The implementation of a communication curriculum will be unsustainable and limited, if the area of human resources and the structures of the organisation are neglected [28].

The development of the "Undeloher recommendation" is not only based on the available literature, but the particular experiences of communication skills experts with difficulties and setbacks. The result was a compilation of over 50 recommendations and key questions based on different phases of the CD from planning to sustainability. At first glance, some of the recommendations seem redundant. These duplications are clarified and adjusted through either the recommendation or the key question according to the particular stage of the process. We recommend to adapt the checklist to the specific context and needs, e.g. if necessary to reduce the number of recommendations. At this point it remains open, whether in the future further key questions about the individual phases of the CD must be supplemented. This can only become apparent with the application and with time.

The application of the "Undeloher recommendation" during the annual meeting of the GMA in Bochum, 2010, revealed that all participants experienced working with the recommendations to be helpful, no matter the stage of the different projects. The format of the in step with the actual practice has been found to be very good and helpful. In particular, the participants experienced the key questions as easily applicable and easy to handle. A prioritisation of the sub-items or putting them in a hierarchy was assessed as rather disadvantageous for the application. The question on how one can best deal with the experience of failure remained open.

A critical subject of debate were the obstacles and challenges emerging working with a "forced group", for example, motivational processes that can have a negative impact on the group dynamics and the cooperation in the group, if participation in the steering committee does not arise from personal motivation. To gain interested non-P-clinicians and pre-clinical to participate on a steering committee to develop a longitudinal curriculum was perceived as a particular challenge and critically discussed. The aim of the existent version of The “Undeloher recommendation” is its dissemination and implementation with the aim of making it available to the experiences of many other experts in curriculum planning.

The limitation of recommendations presented here is that they are hitherto lacking empirical verification. The recommendations have emerged in a long-term discussion process with a variety of teachers. They are based on literature and personal experiences. The extent to which the recommendations have resulted in any particular case to greater sustainability in the implementation of a communication curriculum, has not yet been assessed. At this point the question is whether and to which extent the effectiveness of such a recommendation is at all empirically verifiable, because the different faculties have very different and individual starting conditions and requirements which make a comparison difficult (e.g. traditional vs. reformed curricular, modular courses, semester vs. modules etc.). However, the “Undeloher recommendations” can provide a framework for guidance in the process of curriculum development.

A first step could be retrospective case studies where those who have managed to implement a communication curriculum, check the extent to which they have already applied some of the recommendations and to what extent these have contributed to the success of the project. These results, could then initiate other projects rather prospectively applied in order to verify the quality of the presented recommendations.

## Conclusions

The presented "Undeloher recommendation" has been designed to support the preparation and monitoring of implementation processes of curricula in the field of communication and social skills. It combines results of theoretical models and practical experience of an interdepartmental expert group. It also offers the possibility to analyse ongoing processes that are stalled, and if necessary to steer them in a different direction.

## Acknowledgements

The authors wish to acknowledge the Carl Gustav Carus Foundation (Zürich) for the generous financial support of the workshop in Undeloh and the workshop participants of group C in Undeloh: A. Dieterich (Berlin), E. Gummersbach (Düsseldorf), R. Haak (Köln), P. Jansen (Witten), C. Kiessling (Basel), W. Langewitz (Basel), A. Mortsiever (Düsseldorf), S. Pruskil (Berlin), J.-H. Schultz (Heidelberg). Furthermore we thank G. Fabry (Freiburg) for his valuable comments. We also thank all workshop participants in Undeloh, Bochum and Aachen for their dedicated cooperation and creative suggestions. 

## Competing interests

The authors declare that they have no competing interests. 

## Supplementary Material

Table 3: "Undeloher recommendation" for the sustainable implementation of a longitudinal curriculum for teaching communication and social competencies

## Figures and Tables

**Table 1:**
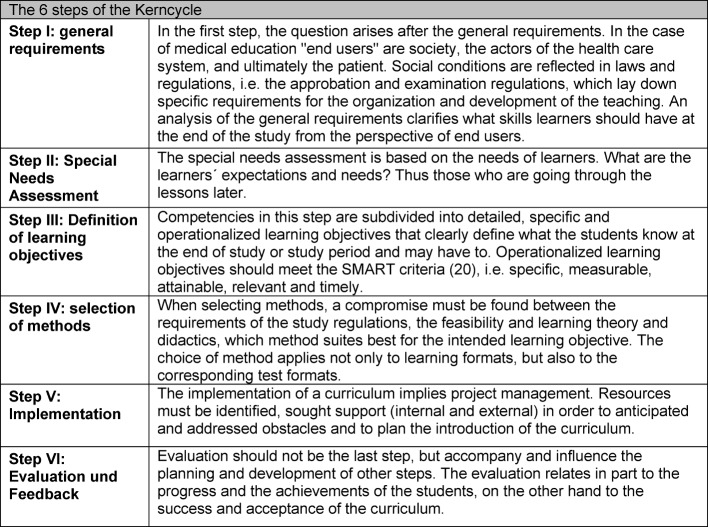
Curriculum development by Kern et al. [13]

**Table 2:**
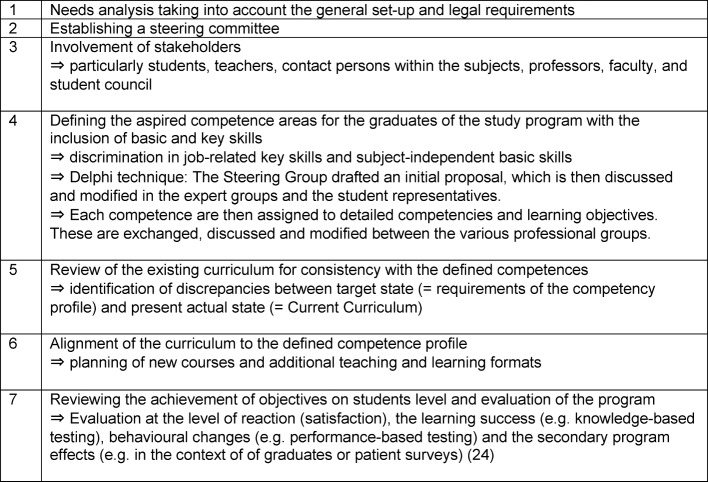
Curriculum development according to Thumser et al. [19]
