# Supplementary material for: Implementation of "social and communicative competencies" in medical education. The importance of curriculum, organisational and human resource development
Source: GMS Z Med Ausbild. 2015 Nov 16;32(5):Doc50. doi: 10.3205/zma000992 (PMC4647157; doi:10.3205/zma000992)
Supplement: Table 3: "Undeloher recommendation" for the sustainable implementation of a longitudinal curriculum for teaching communication and social competencies [file ZMA-32-50-s-001.pdf]

1. Generating a steering committee and organising collaboration (see Thumser Table 2)

Recommendation	Key questions
The first meeting should not be explicitly understood as constitutive of content, but as a group constitution.	Who are we?<br>Where are we going?<br>What do we need to achieve our goals?
Create an active group formation process!	Which skills do we have and do we need?<br>What skills does everyone have and how much time can everyone make available for the group?
Organise your group roles!	Which group roles do we want and need? E.g. management, protocol management, organisation, documentation, external representation, financial planning and management
No start without at least one decision-maker honoured by the Faculty ("Zampano")!	Is there any external / internal recognised decision-maker who would support the project? Who within the faculty may lend weight to the project?
Forget your discipline, but not your daily practice - Think interdisciplinary!	Do the participants understand themselves as group members or as a representative of their specialty?
Integrate "non-P-departments"!	How can we involve the "dedicated non-P-clinicians and pre-clinical"?
Involve students!	When and how is the integration of students useful? e.g. in order to receive information and ideas?<br>Should students be part of the steering committee?
Pay attention to your personal resources	How much time can each person invest for the project?<br>How do we support each other when resources are scarce?
Bear in mind to actively and continuously integrate new members!	Who could / should join?
Develop a preamble or mission statement!	What unites us, what is our vision?<br>What do we want to achieve in concrete terms?
Find a real consensus on the goals! (25)	Do all agree with the formulated objectives?
Define the manner of cooperation and time management (time axis)!	How do we want to cooperate?<br>How mandatory is our co-operation?
Remember to document!	How can we save important documents, so that everyone can access it?
Seek advise at an early stage (e.g. in formulation of objectives, choice of methods, sources, advertising the project)!	Which experts can help us?<br>Whereat do we need help?
Obtain a mandate of the faculty!	What legitimacy does the group have?
Install your group within the structures of the faculty (to avoid foreign body character)!	Where can we position our group within the faculty (in which bodies)?
Culture change takes time!	How do we organise ourselves in order to be active for the years to come?

2. Analysis of the demand, the people involved, the organisation and the environment (see Table 1 Kern I & II and Table 2 Thumser)

Recommendation	Key questions
Create the need!	Is there an awareness of the importance of our concern?<br>How can we establish the need for our project?
Find arguments in support of the project!	What helps us to argue (e.g. licensing regulations, patient surveys, evidence, the demands of society, results of rankings, test results)?
Read the literature!	What is the evidence base?<br>What are the recommendations already?<br>Which supporting and hindering factors have been described in the literature?
Raise the actual status and make an environmental analysis! (see SWOT analysis page 12)	How much space is given for teaching of communication and social skills in the existing curriculum?<br>What activities do already exist?<br>Where are gaps, redundancies, deficits promoting and inhibiting aspects?
Make a stakeholder analysis! (see page 12ff)	Who are the stakeholders?<br>Who are decision-makers?<br>What people promote (groups) or inhibit our plan?
Look out specifically for creative minds, the early converters, the critical silent mass, the resistor and the suspected underground!	Who / what groups can be assigned to these "types" at the Faculty?<br>How can the people be involved, which could be useful for the project?<br>How can we deal with resistance?
Convince the dean's office!	How can the longitudinal curriculum be integrated into the overall training concept of the faculty?<br>What does this mean for the planning and organisation of the overall curriculum?<br>Do we have the support of the teachers in charge?
Make sure you have supporters within the university committees!	Are there people from the university committees that can support and represent the steering group?<br>In which structures of resource allocation (e.g. finance), can the project be inserted?

3. Definition of skills

Recommendation	Key questions
Discuss and formulate common goals!	What do we want to achieve in concrete terms?
Take advantage of an established catalogue of learning objectives (e.g. Basel Consensus Statement) when establishing training objectives for your concept!	Which educational goals are there in total?<br>What are the main educational goals for us?<br>Where do we want to put an emphasis?
With limited resources, focus on the essentials!	What goals are the most important ones for us?<br>What can possibly be still left out?

4. Method selection

Recommendation	Key questions
Plan which teaching methods should be used when and how!	What teaching and learning methods correspond to the learning objectives?<br>What experience is there already with the different methods?<br>What resources do we need for the use of a particular method?
"Keep it simple!"	How can we with the fewest possible resources gain as much as possible?
Seek advice!	What can we learn from others in order to most efficiently use our resources?

5. Implementation

Recommendation	Key questions
Bring the training objectives (and competences) in a chronological sequence over the semester!	What is the quota of hours?<br>Which subjects should be considered?<br>Who does what?
Anchor in the hospital!	What training aims can be taught in a clinical context?
Set a realistic time frame for implementation!	When do we want to achieve what?<br>What milestones are available in this project and when can they be achieved?
Specify a (realistic) time to initialise and aim for a specific initialization of the concept !	When would be a good time to start the project?<br>With what should we start?<br>With which sub-step will we most likely achieve success?
Make a list of the lacked resources!	Who and what do we still need for our target achievement?<br>What resources (e.g. rooms, video cameras, simulated patients) can be used?
Be sure to set quickly attainable goals (also) in order to demonstrate a sense of achievement! (Kotter 8 Steps) (8)	Which interim aim of the project shows us and the parties that we are on the right track?<br>Which intermediate target can be publicly effective?
Try to get an external expert for the public announcement of your plans (sales strategy)!	Who could advertise (external group) our opinion effectively for us?
Be well prepared for the start!	What needs to be prepared, so that the organisation of teaching works? (Toolbox, logistics, contact points, collection of useful addresses)
Put incentives to join in!	How can new teachers be encouraged to participate? (Attributed to teaching duties, a role model for students, publication, opportunity for education research)
Train early and continuously! Sustainability depends especially on the people!	What opportunities exist for staff development? (see HRD page 13ff)

	How many trained teachers do we need at the beginning and in the full expansion of the project?<br>What resources (concept, coach, incentives) we need for training?

6. Assessment & Evaluation

Recommendation	Key questions
Define criteria for success!	How do we know that we have achieved our goals (such as feedback from the students and teachers, students' learning success, changes within the faculty, etc.)?<br>To what extent do the training sessions change the students´ behaviour in the clinical courses/(final year (PJ) etc.?<br>Which results should be communicated to the outside?
Plan from the outset, an assessment plan !	What methods of verification are there?<br>When do we need summative tests and what should they look like?<br>How can we give student feedback?<br>How can we support the learning process through a qualification system?<br>What resources do we have for the assessment?
Pay attention to the assurance of quality (audits, evaluation)! (S. PE S. 13ff)	Who is responsible in the Department of quality assurance?<br>How do we evaluate our courses already?<br>What do we want to evaluate?<br>Do we want to establish accompanying research?

7. Sustainability

Recommendation	Key questions
Make early thoughts on how to sustain the project	Is the control group anchored in the committees of the faculty?<br>Is the longitudinal section curriculum anchored in regular classes?<br>Is our project visible to the public? (Lectures, publications, prices)<br>Is the project financed in the long term?
Anchor support in the university committees!	What people from the Coordination Committee can assist in the university committees and represent the project and the training concept?<br>In which structures of resource allocation (e.g. finance), can the project be inserted?
Mind actively and continuously the integration of new members!	Who could / should still join?<br>Is our group open to new ideas and impulses?<br>What makes our group attractive for new members?
Put incentives to participate	How to motivate new teachers to participate? (Attributed to teaching duties, a role model for students, publication, possibility for training research)?
Be open for advancement of the concept!	How and by whom can new ideas be developed and

	introduced?<br>How do we ensure that ideas and suggestions from students and teachers go into improving the training?<br>How do we stay up-to-date?<br>How do we pass on innovation to our teachers?
Train early and continuously! Sustainability depends especially on the people!	What opportunities exist for staff development?<br>What type of training and mutual support can we offer the teachers?<br>What incentive systems do we have for participating in training?
Document and celebrate the successes!	Marketing! (Lighthouse projects, do good and talk about it) How can we remain visible?<br>Which target groups do we address with our marketing design?<br>Which aspects of the project are particularly suitable for press work?<br>Who benefits from the activities?
Culture change takes time!	How can we support each other if we want to give up?<br>How do we deal with setbacks?<br>How do we deal with important people leaving the project?<br>How do we remain patient and persistent?<br>What gives us strength to stay on board?
